# Integrating nutrition into the mathematics curriculum in Australian primary schools: protocol for a randomised controlled trial

**DOI:** 10.1186/s12937-020-00640-x

**Published:** 2020-11-26

**Authors:** Berit M. Follong, Elena Prieto-Rodriguez, Andrew Miller, Clare E. Collins, Tamara Bucher

**Affiliations:** 1 School of Health Sciences, Faculty of Health and Medicine, The University of Newcastle, Newcastle, NSW Australia; 2 Priority Research Centre for Physical Activity and Nutrition, The University of Newcastle, Newcastle, NSW Australia; 3 Priority Research Centre for Health Behaviour, The University of Newcastle, Newcastle, NSW Australia; 4 School of Education, Faculty of Education and Arts, The University of Newcastle, Newcastle, NSW Australia; 5 School of Environmental and Life Sciences, Faculty of Science, The University of Newcastle, 10 Chittaway Road, Ourimbah, NSW 2258 Australia

**Keywords:** Overweight, Obesity, Prevention, Food skills, Numeracy, Education, Cross-curricular, Healthy eating, Volume estimation, Quality teaching

## Abstract

**Background:**

Nutrition education programs in schools have been effective in improving children’s knowledge and behaviours related to food and nutrition. However, teachers find it challenging to implement such programs due to overcrowded curricula. Integrating nutrition with core subjects such as mathematics could potentially address time constraints and improve the learning of both. The primary aim of this randomized controlled trial (RCT) is to evaluate the impact of a cross-curricular nutrition and mathematics program on primary school students’ portion size estimation skills. Secondary aims include impact on their nutrition knowledge, attitudes towards mathematics and evaluating the quality of the lessons.

**Methods:**

Twelve Year 3–4 classes from Catholic schools in New South Wales, Australia will be randomised to intervention (*n* = 6) or control (*n =* 6) groups. Teachers in the intervention group will receive a professional development workshop and resources to teach 4–5 lessons on portion size and measurements across 1–4 weeks. Outcome measures include portion size estimation skills, nutrition knowledge and attitudes towards mathematics, with data collected during three school visits (pre-intervention, immediately post-intervention, 4 weeks post-intervention). Additionally, teaching quality will be assessed in both intervention and control groups and process evaluation undertaken using teacher interviews and student focus groups.

**Discussion:**

This RCT uses an innovative approach to improve both nutrition and mathematics related learning outcomes among primary school children. It has the potential to impact teaching practices regarding integration of nutrition into curricula and enhance the implementation of nutrition education interventions.

**Trial registration:**

Australian and New Zealand Clinical Trials Register ACTRN12619001071112 31/07/2019.

## Background

Healthy eating patterns promote optimal growth and development throughout infancy and childhood [1]. A healthy diet has benefits for overall well-being, with some studies reporting better school performance [2–4]. Dietary behaviour trends over recent decades show an increase in snacking frequency [5, 6], consumption of sugar-sweetened drinks [7] and larger portion sizes [8] while fruit and vegetable intake have decreased [5]. The latest National Health Survey (2017–18, Australia) recently reported that only 6.6% of New South Wales’ (NSW) children aged 2–17 years met recommendations for daily serves of both fruit and vegetables [9]. Furthermore, almost one-third (30.8%) consumed sugar sweetened drinks on 1–3 days per week. Establishing healthy eating habits during childhood is important as these behaviours tend to track into adulthood [10, 11].

Research indicates that it is not only a matter of what we eat, but also that the amount we consume plays a key role in nutrition-related health [8, 12, 13]. With food portions offered often being supersized, individuals are more likely to consume more food and drinks [14], which increases energy intake [15–17]. Both children and adults perceive serve size recommendations and portion size estimation as confusing [18–21]. Additionally, many people of all ages have difficulties estimating food volumes and portion sizes accurately with multiple studies showing that consumers’ estimations deviate largely from standard serve sizes [19, 22–24]. This might partly explain why large portions offered can lead to an increased consumption, with individuals not stopping once they consume ‘recommended’ amounts of food. Portion size education and training improve individual’s portion size estimation and knowledge [25–27]. Research shows that using (visual) estimation aids can be effective in reducing portion size estimation errors [28–31]. However, the effectiveness of such educational programs and portion size estimation aids in children and adolescents remains unclear [20]. Interventions on portion size education that target this specific population age group are therefore needed.

Schools are an ideal environment to provide nutrition or portion size education. A large number of children can be reached as they are in class during the majority of the week and for a prolonged period of their childhood [32, 33]. Theoretically, teachers have the potential to influence children’s dietary behaviour and knowledge through nutrition education [32, 34]. In practice, teachers experience several barriers to teaching nutrition in the classroom. Barriers such as lack of time and competing demands have been reported frequently [35–43]. Resources are often scarce and education materials are not ‘ready to go’ or not linked to curricular learning outcomes [35, 37, 41, 44]. As a result, difficulties arise when trying to teach nutrition as a sole subject [45] and the teaching of core curricular subjects is prioritised [33, 35, 38, 40]. This highlights the needs for school-based nutrition interventions that involve strategies to eliminate these time-related barriers.

An integrative or cross-curricular approach has been recommended to combat these time constraints faced by teachers [35, 41, 44, 46]. Embedding nutrition into core academic subjects such as Mathematics, English or Science would minimally affect the time spent on these subjects [37, 44, 45, 47]. Subsequently, teachers might be better able to integrate nutrition education programs within existing lessons and be more likely to implement these [44]. Another advantage of integrating nutrition with existing subjects is that it might enhance learning by creating real-life contexts relevant to the students [46–49]. Dudley et al. found that cross-curricular strategies have a positive effect on nutrition knowledge and dietary behaviours amongst schoolchildren [33]. Furthermore, findings from the FoodMASTER initiative, a hands-on mathematics and science curriculum that uses nutrition and food concepts as tools for teaching primary school children, show that a food-based science curriculum improves students’ nutrition [50], mathematics [49] and science knowledge [51].

Similar strategies could be used to integrate portion size education into the primary school curriculum. To accurately estimate portion size, individuals use essential skills including comparison of different amounts of food to other foods, or standard measurement units or estimation aids [52]. Such skills require understanding of basic mathematical concepts [53, 54] including volume and capacity [55]. The link between nutrition and mathematical concepts has been discussed, highlighting that it may be beneficial for development of skills related to both subject areas [48, 53–57]. A recent paper explored teaching practices related to the integration of nutrition and mathematical concepts among Australian primary school teachers. Although teachers reported using nutrition-related examples for their mathematics teaching, the integration of nutrition and mathematics could be substantially improved [55].

To the best of our knowledge, no previous interventions have reported the impact of a classroom-based nutrition education program focussing on portion size estimation nor the integration with mathematics in schools. A newly developed teaching unit incorporates the factors discussed above to maximize its effectiveness in relation to educational outcomes and to optimize implementation in the classroom. This cluster randomised controlled trial (RCT) will investigate the efficacy of a teaching unit integrating nutrition and mathematics in primary school children (CUPS: Cross-curricular Unit on Portion Size).

## Methods/design

### Aim and hypotheses

The overall aim of this RCT is to determine the effect of integrating nutrition into mathematics lessons on primary school children’s portion size estimation skills, nutrition knowledge and attitudes towards mathematics, and to explore teaching quality of the CUPS program.

The research questions for this study are:

1. What is the impact of the CUPS intervention on primary school students’ portion size estimation skills?
2. What is the impact of the CUPS intervention on primary school students’ nutrition knowledge and attitudes towards mathematics?
3. What is the impact of the CUPS intervention on the teaching quality?
4. Is the CUPS intervention feasible for Stage 2 teaching?

It is hypothesized that the CUPS intervention group will show improvements in all outcome measures in comparison to the control group.

### Study setting and design

The CUPS intervention program is a RCT evaluating intervention effectiveness in a primary school setting. Ethics approval has been obtained from the University of Newcastle and the Catholic Diocese of Newcastle-Maitland in NSW, Australia. The CUPS trial is registered with the Australian and New Zealand Clinical Trials Registry (ACTRN12619001071112). The “Standard Protocol Items: Recommendation for Interventional Trials” (SPIRIT) was used to guide design, conduct and reporting of this study (see Additional file 1) [58].

### Recruitment and study participants

The CUPS trial will be conducted in primary schools from the Newcastle and Hunter region of NSW, Australia. Twelve schools from the Catholic Diocese of Newcastle-Maitland will be recruited to participate. From each school, one Stage 2 class (Year 3 and/or 4) will be invited. Students in Stage 2 will be selected as volume and capacity are introduced in this Stage in the NSW K-10 Mathematics syllabus [59]. All 44 Catholic primary schools within the Newcastle-Maitland region will be contacted to invite them into the study. If greater than twelve schools respond to participate, schools will be randomly selected. Principals, teachers, parents and students will need to provide their written informed consent. An email will be sent to the school principals containing Information Statements and Consent Forms. Principals will be followed up by either a reminder email or phone call. Upon receipt of the Principal’s consent, teachers’ Information Statements and informed Consent Forms will be provided to the school to distribute to all Year 3 and/or 4 teachers. To avoid teachers feeling pressured to participate, schools will be asked to send the information through email listings via an administrative staff member. A member of the research team will contact the teachers to organise a school visit and provide students with information about the study. All students wanting to participate in this study will be required to return a Consent Form, which has been signed by the students and their parents/guardians. Signed forms will be returned to the research team via a collection point at the school’s office. All Consent Forms collected through the school’s office will be kept confidential and individual’s privacy will remain protected.

**Fig. 1:**
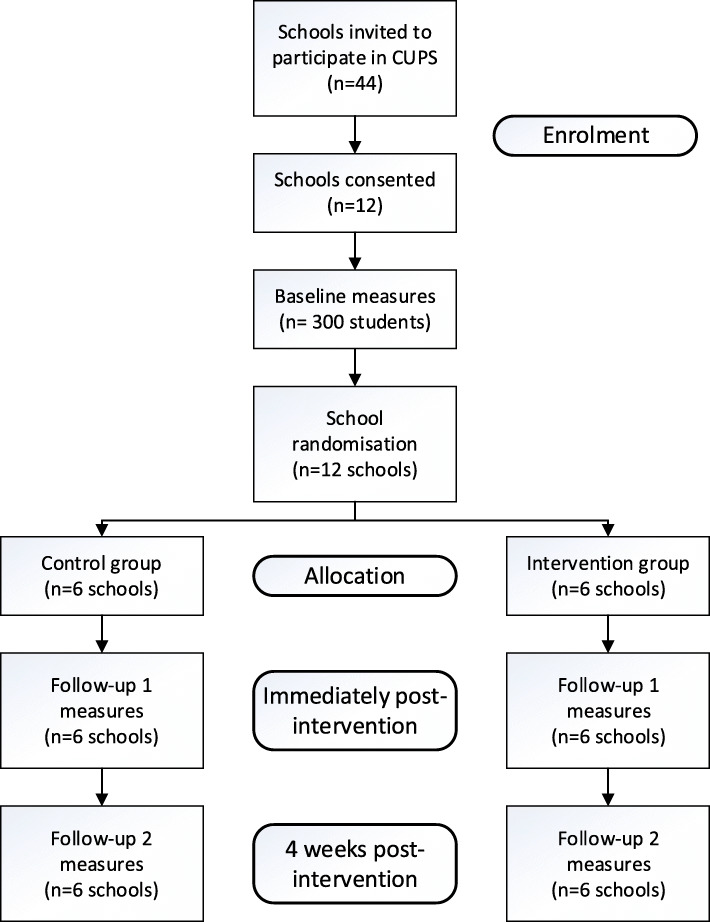
CUPS intervention design including recruitment, group allocation, intervention outline and measurements

Class groups in which the teacher has already taught all nutrition and volume capacity content covered in the planned intervention will be excluded. In addition, teachers with formal nutrition training/professional certification in nutrition or dietetics will be ineligible. All students of consenting teachers will participate in the CUPS program as part of their normal classroom activities. However, only data from students who return their consent letters will be used in analyses. Enrolment of the teachers and their students will be performed by a member of the research team. Figure 1 summarises the participant recruitment and group allocation flowchart.

After baseline measurements, schools will be randomly allocated to either the intervention or wait-list control groups. Participating schools will be matched based on size and socio-economic demographics using the index of community socio-educational advantage (ICSEA). This index produces scores that take into account family background information provided by the families to the schools and includes parental occupation and (non-)school education levels achieved. An independent researcher who is not involved in the CUPS program will use a simple computerised sequence generation to perform the randomisation of the matched schools. Following the randomisation of the schools, a member of the research team will assign the participating schools to either the intervention or the control condition.

### Sample size calculation

A power calculation was conducted to determine the sample size necessary to detect changes in the primary outcome of portion size estimation (% deviance from actual). Based on proportional error reported when using an international food unit [29], detection of a 5% reduction in error was regarded as clinically important. A sample size of 84 was needed to detect a 5% reduction in portion size estimation error (from 20 to 15%) assuming an alpha of 0.05 and power of 80%. To adjust for clustering, the following correction factor was applied [1+ (m – 1) x ICC] [60], where m = students per school and ICC = the intra-class correlation coefficient (between school variance / (between school variance + within school variance). Assumptions are based on clustering at the school level (one class recruited per school, with 25 students per class), and an ICC of 0.1 based on data from a large scale study in Australian schools [61], resulting in a correction factor of 3.4. The resulting student sample is 285 students at 12 schools for a minimum detectable effect size of d = 0.62.

### Intervention

The CUPS intervention will involve the implementation of an integrated teaching unit on nutrition and mathematics. Across one to four weeks, teachers will teach 4–5 lessons, each 40 min in duration, that use a nutrition context to apply learning outcomes of the NSW Mathematics K-10 syllabus [59]. Moreover, the trial will include a three-hour professional development workshop for all teachers and the provision of resources and materials for the schools to keep.

Teachers in the intervention group will be invited to attend the professional development workshop at the University of Newcastle, which will be delivered by researchers from the research team. The research team consists of professionals in the field of Education, Nutrition and Teaching. The workshop is registered and accredited with the NSW Education Standards Authority (NESA) as professional development towards maintaining teaching proficiency. The aim of the workshop is to provide the teachers with the fundamental skills to prepare, plan and implement the CUPS lessons. The workshop will include a rationale for the integration of nutrition into the curriculum, information on the Australian Guide to Healthy Eating (AGHE) [62], introduction to the teaching unit and support with implementation. The AGHE provides evidence-based recommendations about the type of foods and serve sizes individuals should eat for health and overall wellbeing [62]. Attendees will be provided with demonstrations, resources and the latest information to engage students with mathematical and nutrition concepts particularly on volume and capacity, and portion size estimation. They will be introduced to an integrated unit that involves the use of hands-on tools such as mathematics linking cubes and food models. We will discuss the relevance and need for this integrated and experiential approach to learning in primary schools. The AGHE will be used to make teachers familiar with content on healthy eating and portion size estimation. In this workshop, the majority of time will be spent on learning how to integrate the knowledge and skills into the Stage 2 Mathematics curriculum.

Following the completion of the professional development workshop, classes in the intervention group will be given a CUPS school pack containing all information and equipment needed to implement the program. The resources will include food models, mathematics cubes, a set of measuring cups, AGHE posters and brochures, plastic containers, lesson plans, presentation slides and worksheets. The CUPS lessons will specifically focus on learning appropriate portion sizes for each food group and the mathematical concept of volume and capacity. For example, students will learn how to convert volume from the number of mathematics cubes to measuring cups and vice versa. Teaching unit content will be based on the NSW K-10 syllabus and AGHE. Using learning outcomes from both Mathematics [59] and Personal Development, Health and Physical Education (PDHPE) syllabus [63] will enable students to participate in a range of integrated activities. Experienced primary school teachers and researchers in the field of Nutrition and Education developed the lessons involved in this intervention.

The lessons follow a sequence that will teach the students about nutrition concepts on portion/serve sizes in which mathematical concepts on volume and capacity are gradually embedded within each subsequent lesson. This progressive integration of mathematics concepts ensures that the students will become familiar with both subjects in a stepwise manner without being overwhelmed with new information. The first lesson introduces the students to the AGHE including food groups, number of serves per food group and examples of such serve sizes. The students will familiarise themselves with the use of mathematics cubes to estimate portion or serve sizes. In the second lesson, students move from digital examples to visual and hands-on tools such as mathematics linking cubes, measuring cups and food models to identify serve sizes. Students will be provided with the “Healthy eating for children” brochure, which includes the dietary guidelines and serve sizes for children. This lesson requires the use of this brochure for the children to compare, estimate and measure serve sizes based on their own gender and age. A third lesson will involve the comparison of nutrition labels and sugar content of multiple food items. This lesson supports the learning of what information can be found on a nutrition label and how this helps making informed decisions about the healthiness of each food item. In particular, students will identify, estimate and compare the volume of sugar expressed as cubes for the food products. The following lesson builds on the concepts learned in the second lesson in a way that it asks to compare, estimate and measure serve sizes using formal units. Instead of expressing serve or portion sizes in cubes, students will be taught how to convert cubes to cups, cubic centimetres and millilitres. The final lesson assesses students’ understanding of the nutrition and mathematics concepts by having them create a healthy lunchbox. Creating a healthy lunchbox will challenge the student to combine all knowledge and skills gained throughout the previous lessons in regards to food groups, estimating and measuring serve sizes, healthy vs unhealthy foods, and volume and capacity. Additional file 2 summarises the topics and learning outcomes covered by the lessons.

Based on their usual teaching structure, teachers in the intervention group will be asked to plan when they will use the resource material during their classes. As these teachers have to align the CUPS lessons with teaching the mathematics curriculum about “Understanding units of Measurement; volume and capacity”, they will be able to choose when to start with the teaching unit and within what time frame this will be delivered (1–4 weeks). The classroom teacher will provide the lessons to the students during regular teaching time at the primary schools. This ensures that the program will be delivered in a similar way to how it would be delivered outside of the study setting thereby increasing the likelihood of the findings representing a true effect.

Classes allocated to the usual teaching group (wait-list control) will not receive the CUPS program during the study period. Teachers in this wait-list control group will be asked to continue their usual mathematics lessons on volume and capacity. Similarly to the intervention group, teachers in this group will be asked to inform the research team of their plans for teaching volume and capacity and requested not to teach any nutrition-related content. Group comparisons will therefore provide information on the effect of the nutrition integration.

### Outcomes

A variety of both student and teacher related outcome variables will be collected to evaluate the CUPS program effectiveness. Student outcomes include measuring portion size estimation skills, nutrition knowledge, attitudes towards mathematics and their perceptions regarding the CUPS program. In addition, teachers will participate in teaching quality assessments and a semi-structured interview. All activities and assessments will be conducted in a sensitive manner by the research team and trained research assistants. All researchers will receive elaborate instructions prior to assessments to maintain consistency. Variables will be measured during several school visits at baseline, follow-up 1 (1–4 weeks post baseline visit) and follow-up 2 (4 weeks post follow-up 1). Time between data collection at baseline and follow-up visit 1 might differ between schools as teachers are able to decide when to teach the lessons across 1–4 weeks. All schools will be assessed within 1 week after concepts being taught (follow-up 1).

#### Portion size estimation skills

The primary outcome will be students’ portions size estimation skills which will be assessed by determining the ability of a student to estimate the correct portion size of a given food. Mathematics cubes will be used by the children to measure food volume of a set of food models [64]. Researchers will briefly explain the task to the children after which they are asked to perform this themselves. They will be given a variety of food models including an apple, chicken filet pieces, broccoli/cauliflower flowerets, feta cubes and penne pasta. Students will have to compare the volume of both cubes and food models to estimate the number of cubes that correspond with the portion size. The correct food volume expressed as number of cubes will be compared with student answers to determine their ability to estimate portion sizes as percentage deviance from the actual. This methodology has been used in previous research to explore individuals’ portion size estimation skills [29, 65]. Children will also be asked to report the food volume using other units of measurement (e.g. how many serves based on the AGHE). This outcome variable will be taken at all three school visits (baseline, follow-up 1 and follow-up 2).

#### Nutrition knowledge

This secondary outcome measure will be determined using the Child Nutrition Knowledge Survey (CNK-AU) of 57 questions specifically designed for Australian primary school-aged children (de Vlieger et al. manuscript in preparation). This survey is an adapted version of a Belgian nutrition knowledge survey developed by Vereecken et al. [66]. The adaptations include translation into English language and alignment with the Australian dietary guidelines, recommendations and Australian food culture [67]. Common Belgian foods and drinks were replaced by similar products that Australian children are more familiar with based on the Australian Nutrition Survey 2011–2012 [68]. Furthermore, questions about recommended daily serves and food group categorisations were modified to correspond with the AGHE guidelines [62]. This survey has been tested for its reliability in a study with 94 children aged 9 to 12 years by de Vlieger et al. (unpublished observations) observing a moderate to substantial interrater reliability score for most domains (mean κ = 0.43, SD 0.21). Eight domains will be covered to examine nutrition knowledge including, healthy choices (*n* = 11), AGHE serves (*n* = 7), balanced meals (*n* = 6), nutrient & food functions (*n* = 16), food categorisations (*n =* 6), food safety (*n* = 2), nutrition labels (*n =* 2), and food sources (*n =* 7). The CNK-AU consists of 45 multiple-choice questions, 5 multiple answer questions, 4 dichotomous questions and 3 matrix question. Children will earn one point for each correctly answered multiple choice or dichotomous question, each row within a matrix question is worth one point, and each correctly chosen option for multiple answers questions will be scored as one point. As the correct answers regarding the AGHE recommended daily serves depend on a child’s gender and age, point scores will be coded accordingly to adjust for this. Responses including “I don’t know” or multiple answers where only one answer was possible will be scored as incorrect and given zero points. With a total of 82 items, the highest possible score that can be earned is 98 points.

The test will be administered under exam conditions and takes approximately 60 min to complete. Data on this outcome will be collected at all three school visits and will be analysed using overall point scores calculated from each correct answer. The survey will provide evidence of student’s knowledge for specific nutrition related topics and change over time. Alongside the nutrition knowledge survey, data on demographics (i.e., age, gender, school year, cultural background, language, and suburb) and prior nutrition knowledge will be collected via the questionnaire.

#### Attitude towards mathematics

The “How I Feel About Maths Scale” (HIFAMS) has been designed by Chapman [69]. This scale has 10 statements on different aspects of mathematics attitudes (e.g. enjoyment, value and coping). Students will rate their agreement for each statement on a five-point Likert scale ranging from strongly agree to strongly disagree (Table 1). The HIFAMS has specifically been designed for primary school children as a short test using simple language. Students will complete this scale at baseline, follow-up 1 and follow-up 2.

**Table 1:** “How I Feel About Maths Scale” statements and scoring [69]

Statement		Score (*1 = Strongly disagree* to *5 = Strongly agree*)				
1.	Maths is boring	1	2	3	4	5
2.	Maths is too confusing	1	2	3	4	5
3.	I enjoy my maths lessons	1	2	3	4	5
4.	Maths is an important subject	1	2	3	4	5
5.	I can’t keep up with the work we do in maths	1	2	3	4	5
6.	I like maths	1	2	3	4	5
7.	I like maths more than my other school subjects	1	2	3	4	5
8.	Doing maths problems is fun	1	2	3	4	5
9.	I can’t see why I have to do maths	1	2	3	4	5
10.	Maths is a useless subject	1	2	3	4	5

#### Teaching quality

The research team will evaluate the teaching quality of multiple sessions scheduled during the program delivery. In both intervention and control groups, teaching quality related to lessons on volume and capacity will be coded using the Quality Teaching Lesson Observation Scales [70]. These scales are used to evaluate teaching behaviour divided into three dimensions: intellectual quality, quality learning environment and significance of learning. These three dimensions are further subdivided into six elements each (Table 2). Each element contains a descriptive statement which has to be rated on a scale ranging from 1 to 5. The mean of the 18 elements will be used for further statistical analysis [70]. Lessons will be observed and coded by a trained member of the study team. Training sessions will ensure that assessors have experience in this type of coding of lessons. Training includes explanation of the elements, the process of lesson observations and coding, and opportunities to practice coding of several videos which have been previously rated by experts. At least 20% of observations will be joint observation coding to determine inter-rater reliability. These joint observations involve coding discussions between assessors to attain an agreed code [71]. A random sample of participating teachers will be involved in this outcome measure. Three teachers from each study arm will be observed for four lessons which will be scheduled according to the teachers’ timeline.

**Table 2:** Quality Teaching Model [70]

	Intellectual quality	Quality learning environment	Significance
**Elements**	Deep knowledge	Explicit quality criteria	Background knowledge
	Deep understanding	Engagement	Cultural knowledge
	Problematic knowledge	High expectations	Knowledge integration
	Higher-order thinking	Social support	Inclusivity
	Metalanguage	Students’ self-regulation	Connectedness
	Substantive communication	Student direction	Narrative

The Quality Teaching Model evaluates teaching practices and student learning divided into three dimensions: intellectual quality, quality learning environment and significance. Dimension are subdivided into six elements which each describe a statement that has to be rated on a scale ranging from 1 to 5

### Process evaluation

The feasibility and potential of the CUPS intervention will be assessed in the process evaluation using two qualitative research methods. Both teacher and student perspectives on the intervention program will be explored using semi-structured interviews and focus groups, respectively, conducted by members of the research team. These measures will be taken during the school follow-up visit 1.

Interviews with teachers in the intervention group will focus on the program perceptions, barriers and facilitators of the CUPS program implementation and delivery. Teachers will be asked about their experiences with the teaching unit compared to regular mathematics lessons on volume and capacity. The interviews will be designed to gain an insight into major challenges during the implementation of the CUPS lessons, student enjoyment and learning, suggestions for improvements and to obtain feedback on the professional development workshop. Questions from a previous study by Riley et al. (2017) will be modified to the CUPS intervention [72]. For example; Did you enjoy teaching the CUPS lessons as opposed to your usual mathematics lessons? Did you feel confident about teaching the CUPS program? If any, what do you think were the benefits of the CUPS program for you and your students? (See Additional file 3 for all teacher interview questions). This one-hour interview session will be audio recorded and transcribed using a secure transcription service. Teachers will be able to review the recording and/or transcripts of the interview to edit or erase their contribution.

Additionally, researchers will randomly select five students in each class after the intervention finishes to participate in a focus group exploring students’ perceptions on the CUPS program. Only students from the intervention group who have consented to participate in this activity will be invited to join this 30 min session. This methodology has been used previously in a study by Riley et al. (2017) from which we will adapt the focus group questions [72]. Questions will be designed to examine students’ thoughts on the CUPS lessons in comparison with their usual mathematics classes, as well as their opinion on enjoyment regarding both classroom activities and materials, learning outcomes and further improvements of the program (See Additional file 4 for all student focus group questions). The focus groups will be conducted by a member of the research team at the school. Similar to the teacher interview, focus groups will be audio recorded and transcribed by an independent third party.

Any personal information provided by students and teachers will be confidential to the researchers. All participants will be reminded at the beginning of the focus group to maintain the confidentiality of discussion within the setting of the group. Recordings of the interview and focus group will be transcribed by a transcription service which adheres to the Australian Privacy Principles and international equivalents and conforms with university contractor agreements. The results of the study will be published in general terms and will not allow the identification of individual students, teachers or schools. Once the data has been collected, de-identified using participant codes and entered into an electronic data file, questionnaires and other data collection sheets will be destroyed. All databases will be secured with password-protected access systems. The electronic data and audio files will be retained for at least 5 years but no individual will be identifiable in the data files or published reports. The nutrition knowledge survey and portion size estimation protocol can be made available upon request.

### Statistical methods

This intervention is designed to obtain both qualitative and quantitative data. The Statistical Package for the Social Sciences (SPSS) 24 will be used for the data analyses of the primary and secondary outcomes. Descriptive statistics will be explored for all variables including mean, median, standard deviation, and percentages (as data type requires). Differences of means and 95% confidence intervals (CI’s) of quantitative outcomes will be determined using linear mixed models. These models will assess the impact of treatment group (CUPS vs control), time (baseline, follow-up 1 and follow-up 2) and the group-by-time interaction as fixed effects. Covariates in this model will include gender and year level (Year 3 or Year 4). The base model will be further specified by taking clustering of students within classes into account. An alpha level of *p* < 0.05 will be used as cut-off for statistical significance.

Qualitative data from the interviews and focus groups will be transcribed and analysed using a thematic approach. The computer program Leximancer will be used to perform qualitative analyses in a standardised general inductive manner. Leximancer uses automatic content analysis software to visually represent the main concepts from the interviews and focus groups, and to display how these concepts are related [73]. Smith and Humphreys (2006) validated Leximancer using previously published comprehensive evaluation criteria [74]. First, labels or codes derived from the qualitative data will be formulated. Subsequently, these codes will continually be revised and expanded following the coding of additional transcripts. Arising themes will be identified and defined once all transcripts have been coded.

### Dissemination policy

Following the completion of the study, the school will be sent a dissemination report describing the findings of the study. It is suggested that the findings are disseminated to students and their parents/guardians via a school newsletter or similar method. Individual participant data will not be shared with anyone involved in the study or other parties. Furthermore, the data collected from this study will be used to inform future practice for the design of valuable, evidence-based school nutrition programs, through journal publications, conference presentations and in a thesis to be submitted for BMF’s degree. Individual participants will not be identified in any reports arising from the project.

## Discussion

The primary aim of the CUPS RCT is to examine the impact of an integrated nutrition and mathematics program on children’s portion size estimation skills. The secondary aim is to evaluate the effect of the program on student’s nutrition knowledge and attitudes towards mathematics, to determine teaching quality and to explore both teacher’ and student’ perspectives on the intervention. The program uses a strategy to teach children about both portion size and measurements during regular teaching time. Such cross-curricular strategies have been found most effective in improving primary school children’s knowledge and behaviour regarding healthy eating [33].

This teaching concept incorporating learning outcomes related to both the Mathematics and PDHPE strands has the potential to enable teachers to integrate nutrition education into the curriculum. Previous studies have indicated that teachers believe that teaching nutrition is important [41] and that schools play a key role in providing nutrition education [32]. However, research has also highlighted that one of the main barriers for implementing school-based nutrition education is lack of time [35–43]. As the lessons have been specifically designed to align with curricular standards from the NSW K-10 syllabus, teachers are likely to be familiar with some of the concepts, and could justify implementing the program with minimal time lost on teaching core subjects. If found to be effective in improving outcomes, this integrative approach may contribute to implementation of the program more broadly through the reduction of time constrains.

Other commonly reported issues related to not teaching nutrition are no prior nutrition knowledge and the lack of (good quality) resources [35, 41]. Before the start of the intervention period, teachers will be provided with a CUPS program teaching package during a comprehensive professional development workshop. With the professional development workshop, teachers will learn about nutrition, the intervention delivery and have the chance to become familiar with all the concepts. High quality teacher training has been identified as a key factor for improving the impact school-based interventions can make [75] and is critical for providing accurate nutrition information [76, 77]. Teacher professional development has also been shown to improve students learning outcomes [78, 79]. Additionally, each teacher receives a package including the lesson plans, posters, presentation slides, worksheets and other materials. Teachers report that they need resources that are adaptable, from credible sources, aligned with curriculum objectives, low in cost and ‘ready to go’ [35]. Our ‘off the shelf’ resources were developed using an iterative approach and extensive cooperation with teachers to ensure that the lessons are easy to understand and fit within teacher’s usual practices. Findings from multiple studies suggest that provision of both training and adequate instructional resources could significantly improve teachers’ self-efficacy towards teaching nutrition [78, 80]. This is critical, as possessing high self-efficacy is linked to increased time spent teaching nutrition [81] and program implementation [78]. Our approach may support the implementation of the program, as teachers will receive ‘ready to go’ resources that are related to the curriculum and they will be trained to provide nutrition information.

Not only could this integrative intervention improve implementation of nutrition lessons, it may also benefit the students in a way that it increases their engagement and positively influences their attitudes towards mathematics. Enhancing student engagement is particularly essential for mathematics, as previous research has demonstrated that low engagement and negative attitudes can affect students’ mathematics performance [82–84]. It is of great concern that studies often report students expressing negative thoughts towards mathematics [85–87]. Attitudes towards mathematics appear not to be fixed or stable [88], and engagement amongst Australian children has declined [85, 86]. Student enjoyment of mathematics is an essential factor for addressing disengagement [83]. Real-life applications have been suggested as a way to contextualise mathematical concepts [49, 82, 86, 89, 90], support development of in-depth understanding, enhance academic outcomes and learning enjoyment [91]. Research interventions using real-life examples, particularly related to food and nutrition, have shown improvement in mathematics knowledge and achievement [49, 92]. Therefore, our program integrating nutrition and mathematics may positively affect student engagement and attitudes.

This cluster RCT design includes both quantitative and qualitative measures to explore program effectiveness and feasibility. The protocol of the intervention involves detailed process evaluations which are based on previous studies and take teacher’ and student’ perspectives into account. Identifying strengths, barriers and challenges will ensure that nutrition education interventions embedded in academic subjects address teachers’ and students’ needs for sustainable implementation that goes beyond the study duration. The comprehensive process evaluation will help us distinguish between a study that is faulty in its design or poorly delivered, which will be helpful when interpreting the results and for future research [93].

The findings of the CUPS program will provide evidence for the effectiveness of cross-curricular teaching of nutrition and mathematics related subjects and might tackle several barriers to implementing nutrition education. CUPS has the potential to improve student’s portion size estimation skills and knowledge, which are crucial components for positively influencing individuals’ dietary behaviours. Simultaneously, the lessons might enhance student engagement with mathematics and subsequently improve academic performances. Results will inform future research on nutrition education aligned with curricular standards of the mathematics syllabus in primary schools. Classroom based nutrition interventions are infrequent and often lack a detailed description of the integrative approach. CUPS has the potential to influence teaching practices regarding nutrition integration, increase time spent teaching nutrition and enhance a variety of key educational outcomes related to both nutrition and mathematics in primary school children.

## Supplementary Information

**Additional file 1.** SPIRIT Checklist.

**Additional file 2.** CUPS lesson overview including lesson topics, learning intentions and syllabus outcomes.

**Additional file 3.** Teacher interview questions.

**Additional file 4.** Student focus group questions.

## Data Availability

Not applicable.

## Abbreviations

AGHE: Australian Guide to Healthy Eating
CI: Confidence Interval
CUPS: Cross-Curricular Unit on Portion Size
HIFAMS: How I Feel About Maths Scale
ICC: Intra-Class Correlation
ICSEA: Index of Community Socio-Educational Advantage
NESA: NSW Education Standards Authority
NSW: New South Wales
PDHPE: Personal Development, Health and Physical Education
RCT: Randomised Controlled Trial
SPIRIT: Standard Protocol Items: Recommendation for Intervention Trials
SPSS: Statistical Package for the Social Sciences

## References

[1] World Health Organization. Healthy diet 2018. Available from: https://www.who.int/news-room/fact-sheets/detail/healthy-diet. Accessed 3 Feb 2020.

[2] O'Dea JA, Mugridge AC. Nutritional quality of breakfast and physical activity independently predict the literacy and numeracy scores of children after adjusting for socioeconomic status. Health Educ Res. 2012;27(6):975–85. 10.1093/her/cys069 22798563

[3] Correa-Burrows P, Burrows R, Blanco E, Reyes M, Gahagan S (2016). Nutritional quality of diet and academic performance in Chilean students. Bull World Health Organ.

[4] Nyaradi A, Li J, Foster JK, Hickling S, Jacques A, O'Sullivan TA (2016). Good-quality diet in the early years may have a positive effect on academic achievement. Acta Paediatr.

[5] Jahns L, Siega-Riz AM, Popkin BM (2001). The increasing prevalence of snacking among US children from 1977 to 1996. J Pediatr.

[6] Piernas C, Popkin BM (2010). Trends in snacking among US children. Health Aff.

[7] Hardy LL, Grunseit A, Khambalia A, Bell C, Wolfenden L, Milat AJ (2012). Co-occurrence of obesogenic risk factors among adolescents. J Adolesc Health.

[8] Young LR, Nestle M (2002). The contribution of expanding portion sizes to the US obesity epidemic. Am J Public Health.

[9] Australian Bureau of Statistics (2019). Australian Health Survey: Nutrition First Results - Food and Nutrients, 2017–18 Canberra.

[10] Madruga SW, Araújo CLP, Bertoldi AD, Neutzling MB (2012). Tracking of dietary patterns from childhood to adolescence. Rev Saude Publica.

[11] Craigie AM, Lake AA, Kelly SA, Adamson AJ, Mathers JC (2011). Tracking of obesity-related behaviours from childhood to adulthood: a systematic review. Maturitas..

[12] Livingstone MBE, Pourshahidi LK (2014). Portion size and obesity. Adv Nutr.

[13] Hetherington MM (2019). The portion size effect and overconsumption–towards downsizing solutions for children and adolescents–an update. Nutr Bull.

[14] Hollands GJ, Shemilt I, Marteau TM, Jebb SA, Lewis HB, Wei Y, et al. Portion, package or tableware size for changing selection and consumption of food, alcohol and tobacco. Cochrane Database Syst Rev. 2015;9. 10.1002/14651858.CD011045.pub2 PMC4579823 26368271

[15] Wansink B (2004). Environmental factors that increase the food intake and consumption volume of unknowing consumers. Annu Rev Nutr.

[16] Rolls BJ, Roe LS, Meengs JS (2006). Larger portion sizes lead to a sustained increase in energy intake over 2 days. J Am Diet Assoc.

[17] Rolls BJ, Roe LS, Meengs JS (2007). The effect of large portion sizes on energy intake is sustained for 11 days. Obesity (Silver Spring).

[18] Spence M, Livingstone MBE, Hollywood LE, Gibney ER, O’Brien SA, Pourshahidi LK (2013). A qualitative study of psychological, social and behavioral barriers to appropriate food portion size control. Int J Behav Nutr Phys Act.

[19] Collins CE, Bucher T, Taylor A, Pezdirc K, Lucas H, Watson J (2015). How big is a food portion? A pilot study in Australian families. Health Promot J Austr.

[20] Bucher T, Rollo ME, Smith SP, Dean M, Brown H, Sun M (2017). Position paper on the need for portion-size education and a standardised unit of measurement. Health Promot J Austr.

[21] Faulkner GP, Pourshahidi LK, Wallace JM, Kerr MA, McCrorie TA, Livingstone MBE (2012). Serving size guidance for consumers: is it effective?. Proc Nutr Soc.

[22] Nørnberg TR, Houlby L, Jørgensen LN, He C, Pérez-Cueto FJA (2014). Do we know how much we put on the plate? Assessment of the accuracy of self-estimated versus weighed vegetables and whole grain portions using an intelligent buffet at the FoodScape lab. Appetite..

[23] Almiron-Roig E, Solis-Trapala I, Dodd J, Jebb SA (2013). Estimating food portions. Influence of unit number, meal type and energy density. Appetite..

[24] Slawson DL, Eck LH (1997). Intense practice enhances accuracy of portion size estimation of amorphous foods. J Acad Nutr Diet.

[25] Small L, Lane H, Vaughan L, Melnyk B, McBurnett D (2013). A systematic review of the evidence: the effects of portion size manipulation with children and portion education/training interventions on dietary intake with adults. Worldviews Evid-Based Nurs.

[26] Steenhuis IHM, Vermeer WM (2009). Portion size: review and framework for interventions. Int J Behav Nutr Phys Act.

[27] Steenhuis I, Poelman M (2017). Portion size: latest developments and interventions. Curr Obes Rep.

[28] Byrd-Bredbenner C, Schwartz J (2004). The effect of practical portion size measurement aids on the accuracy of portion size estimates made by young adults. J Hum Nutr Diet.

[29] Bucher T, Weltert M, Rollo ME, Smith SP, Jia W, Collins CE (2017). The international food unit: a new measurement aid that can improve portion size estimation. Int J Behav Nutr Phys Act.

[30] English L, Lasschuijt M, Keller KL (2015). Mechanisms of the portion size effect. What is known and where do we go from here?. Appetite..

[31] Steyn NP, Senekal M, Norris SA, Whati L, MacKeown JM, Nel JH (2006). How well do adolescents determine portion sizes of foods and beverages?. Asia Pac J Clin Nutr.

[32] Clarke J, Fletcher B, Lancashire E, Pallan M, Adab P (2013). The views of stakeholders on the role of the primary school in preventing childhood obesity: a qualitative systematic review. Obes Rev.

[33] Dudley DA, Cotton WG, Peralta LR (2015). Teaching approaches and strategies that promote healthy eating in primary school children: a systematic review and meta-analysis. Int J Behav Nutr Phys Act.

[34] Metos JM, Sarnoff K, Jordan KC (2019). Teachers' perceived and desired roles in nutrition education. J Sch Health.

[35] Love P, Booth A, Margerison C, Nowson C, Grimes C. Food and nutrition education opportunities within Australian primary schools. Health Promot Int. 2020. 10.1093/heapro/daz132 31951256

[36] Totura CM, Figueroa HL, Wharton C, Marsiglia FF (2015). Assessing implementation of evidence-based childhood obesity prevention strategies in schools. Prev Med Rep.

[37] Jones AM, Zidenberg-Cherr S (2015). Exploring nutrition education resources and barriers, and nutrition knowledge in teachers in California. J Nutr Educ Behav.

[38] Upton P, Taylor C, Upton D (2012). Exploring primary school teachers' experiences of implementing a healthy eating intervention. Educ Health.

[39] Pittman DW, Bland IR, Cabrera ID, Franck KE, Perkins EL, Schmidt NA, et al. The boss’ healthy buddies nutrition resource is effective for elementary school students. J Obes. 2018;1-10. 10.1155/2018/4659874 PMC5954886 29854438

[40] Hall E, Chai W, Albrecht JA (2016). A qualitative phenomenological exploration of teachers' experience with nutrition education. Am J Health Educ.

[41] de Vlieger N, Riley N, Miller A, Collins CE, Bucher T. Nutrition education in the Australian New South Wales primary school curriculum: an exploration of time-allocation, translation and attitudes in a sample of teachers. Health Promot J Austr. 2018;7(4):24. 10.1002/hpja.188 30054958

[42] Perikkou A, Kokkinou E, Panagiotakos DB, Yannakoulia M (2015). Teachers’ readiness to implement nutrition education programs: beliefs, attitudes, and barriers. J Res Child Educ.

[43] Prelip M, Erausquin JT, Slusser W, Vecchiarelli S, Weightman H, Lange L (2006). Role of classroom teachers in nutrition and physical education. Californian J Health Promot.

[44] Peralta LR, Dudley DA, Cotton WG (2016). Teaching healthy eating to elementary school students: a scoping review of nutrition education resources. J Sch Health.

[45] Perera T, Frei S, Frei B, Wong SS, Bobe G (2015). Improving nutrition education in US elementary schools: challenges and opportunities. J Educ Pract.

[46] Carraway-Stage V, Kolasa KM, Díaz SR, Duffrin MW (2018). Exploring the associations among nutrition, science, and mathematics knowledge for an integrative, food-based curriculum. J Sch Health.

[47] Miller M (2014). Nutrition literacy needs cross-curriculum learning: medical Xpress.

[48] James DC, Adams TL (1998). Curriculum integration in nutrition and mathematics. J Sch Health.

[49] Roseno AT, Carraway-Stage VG, Hoerdeman C, Díaz SR, Geist E, Duffrin MW (2015). Applying mathematical concepts with hands-on, food-based science curriculum. Sch Sci Math.

[50] Carraway-Stage V, Hovland J, Showers C, Díaz S, Duffrin MW (2015). Food-based science curriculum yields gains in nutrition knowledge. J Sch Health.

[51] Hovland JA, Carraway-Stage VG, Cela A, Collins C, Díaz SR, Collins A (2013). Food-based science curriculum increases 4th graders multidisciplinary science knowledge. J Food Sci Educ.

[52] Trucil LM, Vladescu JC, Reeve KF, DeBar RM, Schnell LK (2015). Improving portion-size estimation using equivalence-based instruction. Psychol Rec.

[53] Hyman B (2008). Integrating math and nutrition education: teaching with the FDA food label. Am J Health Educ.

[54] Weber JL, Cunningham-Sabo L, Skipper B, Lytle L, Stevens J, Gittelsohn J (1999). Portion-size estimation training in second- and third-grade American Indian children. Am J Clin Nutr.

[55] Follong BM, Prieto-Rodriguez E, Miller A, Collins CE, Bucher T (2020). An exploratory survey on teaching practices integrating nutrition and mathematics in Australian primary schools. Int J Res Educ Sci.

[56] Pérez-Rodrigo C, Aranceta J (2003). Nutrition education in schools: experiences and challenges. Eur J Clin Nutr.

[57] Duffrin MW, Cuson D, Phillips SK (2005). Using food to boost math and science skills. J Fam Consum Sci.

[58] Chan A-W, Tetzlaff JM, Altman DG, Laupacis A, Gøtzsche PC, Krleža-Jerić K (2013). SPIRIT 2013 statement: defining standard protocol items for clinical trials. Ann Intern Med.

[59] Education Standards Authority NSW (2012). Mathematics K-10 syllabus.

[60] Donner A, Klar N, Klar NS (2000). Design and analysis of cluster randomization trials in health research.

[61] Lamb S, Fullarton S (2001). Classroom and school factors affecting mathematics achievement: a comparative study of the US and Australia using TIMSS.

[62] National Health and Medical Research Council. Australian Dietary Guidelines Educator Guide. Canberra: National Health and Medical Research Council; 2013.

[63] NSW Education Standards Authority. Personal development, health and physical Education K–10 syllabus. Sydney: NSW Education Standards Authority; 2018.

[64] Bucher T, van der Horst K, Siegrist M (2012). The fake food buffet – a new method in nutrition behaviour research. Br J Nutr.

[65] de Vlieger NM, Weltert M, Molenaar A, McCaffrey TA, Rollo ME, Truby H (2020). A systematic review of recall errors associated with portion size estimation aids in children. Appetite..

[66] Vereecken C, De Pauw A, Van Cauwenbergh S, Maes L (2012). Development and test–retest reliability of a nutrition knowledge questionnaire for primary-school children. Public Health Nutr.

[67] de Vlieger N, van Rossum J, Riley N, Miller A, Collins C, Bucher T (2020). Nutrition Education in the Australian New South Wales primary school curriculum: knowledge and attitudes of students and parents. Children (Basel).

[68] Australian Bureau of Statistics. Australian Health Survey: Nutrition First Results - Food and Nutrients, 2011–12 Canberra; 2014. https://www.abs.gov.au/statistics/health/health-conditions-and-risks/australian-health-survey-nutrition-first-results-foods-and-nutrients/latest-release. Accessed 24 June 2020.

[69] Chapman E. Development and validation of a brief mathematics attitude scale for primary-aged students. J Educ Enq. 2003;4(2):63-73.

[70] NSW Department of Education and Training (2006). Quality teaching in NSW public schools: a classroom practice guide.

[71] Miller A, Gore J, Wallington C, Harris J, Prieto-Rodriguez E, Smith M (2019). Improving student outcomes through professional development: protocol for a cluster randomised controlled trial of quality teaching rounds. Int J Educ Res.

[72] Riley N, Lubans DR, Holmes K, Hansen V, Gore J, Morgan PJ (2017). Movement-based mathematics: enjoyment and engagement without compromising learning through the EASY minds program. Eurasia J Math Sci Technol Educ.

[73] Smith AE (2018). Leximancer user guide: Release 4.5.

[74] Smith AE, Humphreys MS (2006). Evaluation of unsupervised semantic mapping of natural language with Leximancer concept mapping. Behav Res Methods.

[75] Avalos B (2011). Teacher professional development in teaching and teacher education over ten years. Teach Teach Educ.

[76] Sadegholvad S, Yeatman H, Parrish AM, Worsley A (2017). Experts’ views regarding Australian school-leavers’ knowledge of nutrition and food systems. Aust N Z J Public Health.

[77] Nanayakkara J, Margerison C, Worsley A. Teachers’ perspectives of a new food literacy curriculum in Australia. Health Educ. 2018;118(1):48-61.

[78] Fahlman M, McCaughtry N, Martin J, Shen B (2011). Efficacy, intent to teach, and implementation of nutrition education increases after training for health educators. Am J Health Educ.

[79] Ross JG, Luepker RV, Nelson GD, Saavedra P, Hubbard BM (1991). Teenage health teaching modules: impact of teacher training on implementation and student outcomes. J Sch Health.

[80] Carraway-Stage V, Roseno A, Hodges CD, Hovland J, Diaz S, Duffrin MW (2016). Implementation of a food-based science curriculum improves fourth-grade educators’ self-efficacy for teaching nutrition. Am J Health Educ.

[81] Britten P, Lai MK (1998). Structural analysis of the relationships among elementary teachers’ training, self-efficacy, and time spent teaching nutrition. J Nutr Educ.

[82] Australian Curriculum Assessment and Reporting Authority (2009). Shape of the Australian curriculum: Mathematics. Australian Curriculum, Assessment and Reporting Authority.

[83] Martin AJ, Anderson J, Bobis J, Way J, Vellar R (2012). Switching on and switching off in mathematics: an ecological study of future intent and disengagement among middle school students. J Educ Psychol.

[84] Greenwood CR, Horton BT, Utley CA (2002). Academic engagement: current perspectives on research and practice. Sch Psychol Rev.

[85] Attard C (2013). “If I had to pick any subject, it wouldn’t be maths”: foundations for engagement with mathematics during the middle years. Math Educ Res J.

[86] Attard C (2011). “My favourite subject is maths. For some reason no-one really agrees with me”: student perspectives of mathematics teaching and learning in the upper primary classroom. Math Educ Res J.

[87] Larkin K, Jorgensen R (2015). ‘I hate maths: why do we need to do maths?’ Using iPad video diaries to investigate attitudes and emotions towards mathematics in year 3 and year 6 students. Int J Sci Math Educ.

[88] Pierce R, Stacey K, Barkatsas A (2007). A scale for monitoring students’ attitudes to learning mathematics with technology. Comput Educ.

[89] Sullivan P. Teaching mathematics: using research-informed strategies. Camberwell: Australian Council for Educational Research; 2011.

[90] Wilkinson L, Sullivan P, Jorgensen R (2009). 2009 futureSACE school to work innovation program: literacy & numeracy project final report.

[91] Ladwig J, King M (2003). Quality teaching in NSW public schools: an annotated bibliography.

[92] Shilts MK, Lamp C, Horowitz M, Townsend MS (2009). Pilot study: EatFit impacts sixth graders' academic performance on achievement of mathematics and English education standards. J Nutr Educ Behav.

[93] Oakley A, Strange V, Bonell C, Allen E, Stephenson J (2006). Process evaluation in randomised controlled trials of complex interventions. BMJ..

