# Supplementary material for: Integrating nutrition into the mathematics curriculum in Australian primary schools: protocol for a randomised controlled trial
Source: Nutr J. 2020 Nov 26;19:128. doi: 10.1186/s12937-020-00640-x (PMC7694306; doi:10.1186/s12937-020-00640-x)
Supplement: Supplementary file 2 — Additional file 2. CUPS lesson overview including lesson topics, learning intentions and syllabus outcomes. [file 12937_2020_640_MOESM2_ESM.pdf]

CUPS Lesson overview

<table>
<tr><th>Lesson #</th><th>Lesson Topic</th><th>Learning Intention</th><th>Syllabus Outcome(s)</th></tr>
<tr><td>1</td><td>Introduction to Food Groups and Serve Sizes</td><td>• Identify food groups
• Identify a serve size
• Estimate and measure a variety of foods
• Compare different quantities of food</td><td rowspan="2"><u>Mathematics:</u>

Communicating
MA2-1WM uses appropriate terminology to describe, and symbols to represent, mathematical ideas

Volume and Capacity
MA2-11MG measures, records, compares and estimates volumes and capacities using litres, millilitres and cubic centimetres

<u>Personal Development and Health:</u>
PD2-6 describes how contextual factors are interrelated and how they influence health, safety, wellbeing and participation in physical activity</td></tr>
<tr><td>2</td><td>Introduction to Estimating Serve Sizes using Food models and Cubes</td><td>• Revise a portion
• Be able to use cubes and food models to compare and estimate serve sizes
• Estimate and measure the correct serve size of a variety of foods from a variety of food groups
• Identify how many serves of each food group is recommended daily</td></tr>
<tr><td>3</td><td>Nutritional Labels & Sugar Content</td><td>• Identify the types of information found on a nutrition label
• Identify which pieces of information can help us to make decisions about what we eat and how much we eat
• Identify, estimate and compare the volume of sugar in several food products</td><td><u>Mathematics:</u>

Communicating
MA2-1WM uses appropriate terminology to describe, and symbols to represent, mathematical ideas

Volume and Capacity
MA2-11MG measures, records, compares and estimates volumes and capacities using litres, millilitres and cubic centimetres

Data
MA2-18SP selects appropriate methods to collect data, and constructs, compares, interprets and evaluates data displays, including tables, picture graphs and column graphs</td></tr>
</table>

			Personal Development and Health:<br>PHS2-12 Discusses the factors influencing personal health choices
**4**	Formal Units of Measurement: Cups	• Understand that a portion size expressed in "cubes" can be measured in a formal unit<br>• Convert cubes to cups, millilitres, cubic centimetres and back<br>• Identify a serve size of a particular food and estimate what that is in cups, millilitres, cubic centimetres	***Mathematics:***<br><br>***Communicating***<br>MA2-1WM uses appropriate terminology to describe, and symbols to represent, mathematical ideas<br><br>***Volume and Capacity***<br>MA2-11MG measures, records, compares and estimates volumes and capacities using litres, millilitres and cubic centimetres<br><br>***Personal Development and Health:***<br>PHS2-12 Discusses the factors influencing personal health choices
**5**	Assessment: Creating a Healthy Lunchbox	• Create lunch boxes that have positive food choices in relation to food serve sizes<br>• Explain their reasoning for choosing certain food types and amounts	***Mathematics:***<br><br>***Communicating***<br>MA2-1WM uses appropriate terminology to describe, and symbols to represent, mathematical ideas<br><br>***Volume and Capacity***<br>MA2-11MG measures, records, compares and estimates volumes and capacities using litres, millilitres and cubic centimetres<br><br>***Personal Development and Health:***<br>PD2-6 describes how contextual factors are interrelated and how they influence health, safety, wellbeing and participation in physical activity<br><br>PHS2-12 Discusses the factors influencing personal health choices
