# Supplementary material for: Integrating nutrition into the mathematics curriculum in Australian primary schools: protocol for a randomised controlled trial
Source: Nutr J. 2020 Nov 26;19:128. doi: 10.1186/s12937-020-00640-x (PMC7694306; doi:10.1186/s12937-020-00640-x)
Supplement: Supplementary file 3 — Additional file 3. Teacher interview questions. [file 12937_2020_640_MOESM3_ESM.pdf]

Teacher interview questions

Context

- What is your current teaching role at this school?
  - Permanent/casual
  - Fulltime/part-time
  - Year level
- What is your teaching background/experience?
  - Number of years
  - Localities/contexts
  - Specialist areas
  - Year levels
  - Nutrition education experience
- Can you describe the current school/class context in relation to nutrition education:
  - Timetabling
  - Resources: Facilities, equipment and human support
- Is nutrition education valued at this school? Why/Why not? What makes you think this?
- Do you value nutrition education? Why or why not?
- What aspects of the school context hinder/facilitate teaching of nutrition education?

Program evaluation

- Did you enjoy the professional development? Why or why not?
- Has this professional development been beneficial for you? Why or why not?

- Did you teach all lessons included in the CUPS program? If not, why?
- Can you tell me about your experiences with the CUPS program?
- Did you enjoy teaching the CUPS lessons as opposed to your usual mathematics lessons? How would you rate this on a scale from one to five (1= not enjoyable at all, 2= slightly unenjoyable, 3=neutral, 4=slightly enjoyable, 5=very enjoyable)
- Did you feel confident about teaching the CUPS program? Why or why not? How would you rate this on a scale from one to five (one being not confident at all and five being very confident)
- What were the major challenges to you as a teacher of the CUPS program?
- Did this cross-curricular approach influence any of the previously mentioned barriers to teach nutrition education? Why or why not?
- Do you think your students enjoyed the lessons? Why or why not? Have you noticed any changes with your students? How would you rate this on a scale from one to five (one being not enjoyable at all and five being very enjoyable).
- If any, what do you think were the benefits of the CUPS program for you and your students?
- How well do you think the students understood the mathematics content in the nutrition lessons? Can you give me a specific example?
- Do you think the nutrition aspect of the lesson contributed to greater engagement in the lesson compared to how mathematical content would usually be taught?
- Do you think that the CUPS sufficiently supports the learning of nutritional concepts/mathematical concepts? How would you rate this on a scale from one to five (one being not supportive at all and five being very supportive).

- Is there anything that could be changed to improve the CUPS program?
- What was the best thing about being involved in this CUPS program?

- Are you likely to continue with this cross-curricular approach after the study? Why or why not?
- Are you likely to use a similar cross-curricular approach when teaching other subjects? Why or why not?
- Have you disseminated this information to other staff members?
- How did students contribute to the lesson activities? Was this successful?
- Do you have anything else to say about the CUPS program?
