# Supplementary material for: Integrating nutrition into the mathematics curriculum in Australian primary schools: protocol for a randomised controlled trial
Source: Nutr J. 2020 Nov 26;19:128. doi: 10.1186/s12937-020-00640-x (PMC7694306; doi:10.1186/s12937-020-00640-x)
Supplement: Supplementary file 4 — Additional file 4. Student focus group questions. [file 12937_2020_640_MOESM4_ESM.pdf]

Student focus group questions

- Can you tell me what the CUPS program is all about?
- How would you describe your maths classes before the CUPS program? Did you enjoy this?
- Did you enjoy the lessons in the CUPS program? Why? Can you give me an example? How would you rate this on a scale from one to five (one being not enjoyable at all and five being very enjoyable).
- Did you enjoy the food models in the classroom? And the maths linking cubes? Did this make the maths activities more interesting? How would you rate this on a scale from one to five (one being not enjoyable at all and five being very enjoyable).
- What kinds of activities did you enjoy doing in the CUPS program?
- What kinds of activities didn't you enjoy doing in the CUPS program?
- Can you tell me if the real-life nutrition topics in math class helped you learn? Why/why not? If so, can you give me an example? Was it fun and enjoyable?
- Do you feel you have more knowledge on nutrition and healthy eating after the CUPS lessons? What do you know now what you didn't know before the CUPS program?

- What was the best thing about being involved in the CUPS program?
- Is there anything that could be changed to make the CUPS program better?
- Do you have anything else to say about the CUPS program?
